# Design of liquid level detection circuit based on sampling probe structure capacitance

**DOI:** 10.1371/journal.pone.0298282

**Published:** 2024-04-18

**Authors:** Zeyu Ding, Wei Gong

**Affiliations:** 1 Department of Computer, Changzhi University, Changzhi, Shanxi, China; 2 School of Medical Information Engineering, Guangzhou Univ Chinese Med, Guangzhou, Guangdong, China; Vellore Institute of Technology, INDIA

## Abstract

Liquid level detection system is an essential core functional component of automatic clinical medical testing instrument. The conventional liquid level detection method has low detection accuracy and sensitivity, and may have the problem of false detection, which may lead to the inaccurate test results. This paper presents a high sensitivity liquid level detection system based on the principle of variable capacitance. When the sampling probe contacts the liquid level, the probe capacitance will change. The liquid level detection circuit board judges whether the probe contacts the liquid level by sensing the change of probe capacitance. When judging the liquid level signal, the combination of slope detection and amplitude detection is used. The liquid level detection circuit board takes the phase-locked loop(PLL) circuit as the center to detect the change of the capacitance. The reference signal of the PLL is set as a square wave of 375kHz. The double tube probe is used as a part of the tuning capacitor of the voltage controlled oscillator to control the frequency of the output signal, which can realize the rapid phase locking. The experimental results show that the system has accurate detection results, high sensitivity, stable and reliable operation, good dynamic response performance in the case of large and small liquid volume. Compared with other liquid level detection methods based on machine vision, ultrasonic, optics and so on, the proposed liquid level detection system has simpler structure and lower cost, it can avoid the problems of collision, carryover contamination and empty suction by controlling the depth of sampling needle inserted into liquid.

## 1 Introduction

With the development of biotechnology and electronic technology, clinical automatic medical testing instruments have developed from semi-automation to automation, from low speed to high speed [1]. As an extremely important part of the automatic medical testing instrument, the accuracy and precision of the automatic sampling module seriously affect the accuracy of the test results, and the liquid level detection system is an indispensable core functional component of the automatic sampling module [2, 3].The liquid level detection system controls the depth of the sampling probe inserted into the liquid to avoid the phenomenon of "hitting the probe" or carryover contamination due to the too deep insertion of the sampling probe, or the phenomenon of "empty suction" due to the inability to absorb enough samples or reagents due to the too shallow insertion.

The detection methods of liquid level detection system are different, but they can be summarized into the following detection principles [4–9]:

①Based on the mechanical principle, that is, the force on the sensing element is directly proportional to the liquid level, and the force is converted into the corresponding liquid level height.

②Based on the principle of relative change, that is, when the liquid level changes, the distance between the liquid level and the bottom or top of the container changes, and the information of the liquid level is obtained by measuring the relative change of the distance. This kind of detection principle includes acoustic, microwave, optics, etc.

③Based on the principle that an intensity physical quantity changes with the change of liquid level, the information of liquid level is obtained, such as the absorption intensity of rays, the electrode area or dielectric constant of the capacitor, etc.

At present, the existing liquid level detection technology can be generally divided into two categories: contact and non-contact. Contact type refers to only when the sampling needle comes into contact with the liquid, a way to detect the presence of liquid and subsequently detect the height of the liquid surface. Common contact liquid level detection technologies include resistance, capacitance, pneumatic, resonance methods, etc. Non-contact type refers to a method in which the sampling needle can sense the presence of a liquid without touching it, thereby detecting the height of the liquid surface. Common non-contact liquid level detection technologies include ultrasound, laser and imaging methods.

Resistance method refers to the downward movement of two metal electrodes installed on the sampling needle when they come into contact with a conductive liquid, the resistance between electrodes suddenly decreases. ‚ This method is ineffective for liquids with poor conductivity and requires the appropriate spacing between the two electrodes. Too far apart, not suitable for small caliber tubes; being too close together can form a liquid film between the two electrodes.

The pneumatic method is not affected by the conductivity of liquids and the sampling needle does not need to be made of metal either. When the sampling needle moves downwards under the drive of the transmission mechanism, the needle cylinder piston moves slowly upwards to blow out the air inside the needle cylinder through the sampling needle. Once the sampling needle contacts the liquid level, air output obstructed, the air pressure inside the pipeline increases, detected by the pressure sensor. This measurement method requires the use of high-precision, flush pressure sensors, which need to be continuously calibrated during the measurement process, and repeated calibration is required for long-term use or liquid replacement.

When the sampling needle system resonates mechanically under external forces, once in contact with liquid, the natural frequency of the system changes. Detecting this change can detect whether the sampling needle is in contact with the liquid level. The resonance method is suitable for the case where the liquid surface has foam and the sample tube has a rubber plug, but the system structure is complex.

The principle of ultrasonic measurement of liquid level is that the ultrasonic pulse signal emitted by the ultrasonic probe propagates in the gas and is reflected upon encountering the interface between air and liquid. After receiving the echo signal, the propagation time of the ultrasonic wave back and forth is calculated, and the distance or liquid level height can be converted.

The principle of laser method for measuring liquid level is to fix the laser and photosensitive detector on both sides of the sampling needle and form a certain angle with the sampling needle. Only when the sampling needle is moved down to a fixed height from the liquid surface, can the laser be reflected by the liquid surface and precisely illuminate the photodetector. Based on this, the liquid level height can be detected.

The imaging method is achieved by capturing images of sample tubes to identify the height of the liquid level from the image.

The non-contact liquid level detection technology has problems such as high system cost, relatively complex structure, and susceptibility to interference, so it has not been widely used in practical systems.

This paper presents a high sensitivity liquid level detection system based on the principle of variable capacitance. The system uses a high-reliability capacitive liquid level sensor to convert the liquid level information into the change of sensing capacitance, and uses a phase-locked loop(PLL) circuit to detect the change of capacitance. PLL circuit is the core part of the liquid level detection system, its main functions include:①The information of capacitance change during the movement of sampling probe is transformed into the output frequency change of PLL voltage controlled oscillator (VCO).②Compare the output signal of VCO with the reference signal to output the phase error signal.③Output DC error signal by low-pass filtering of error signal.④The DC error signal is fed back to the VCO, and then the output frequency of the VCO is adjusted to realize the lock of the PLL. The test results show that the system has simple structure, good dynamic response, high sensitivity and good application prospect.

## 2 System design scheme

**Fig 1:**
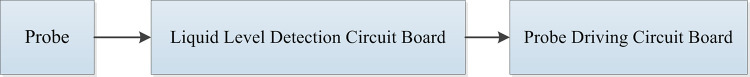
Schematic diagram of liquid level detection system.

The liquid level detection adopts the contact capacitance, that is, the sampling probe is taken as a generalized capacitance. When the probe contacts the liquid level, the probe capacitance will change significantly. The liquid level detection circuit board judges whether the probe contacts the liquid level by sensing the change of probe capacitance, and outputs the liquid level signal to the probe driving circuit board after signal processing. The schematic diagram of the system is shown in Fig 1.

### 2.1 Structure of sampling probe

**Fig 2:**
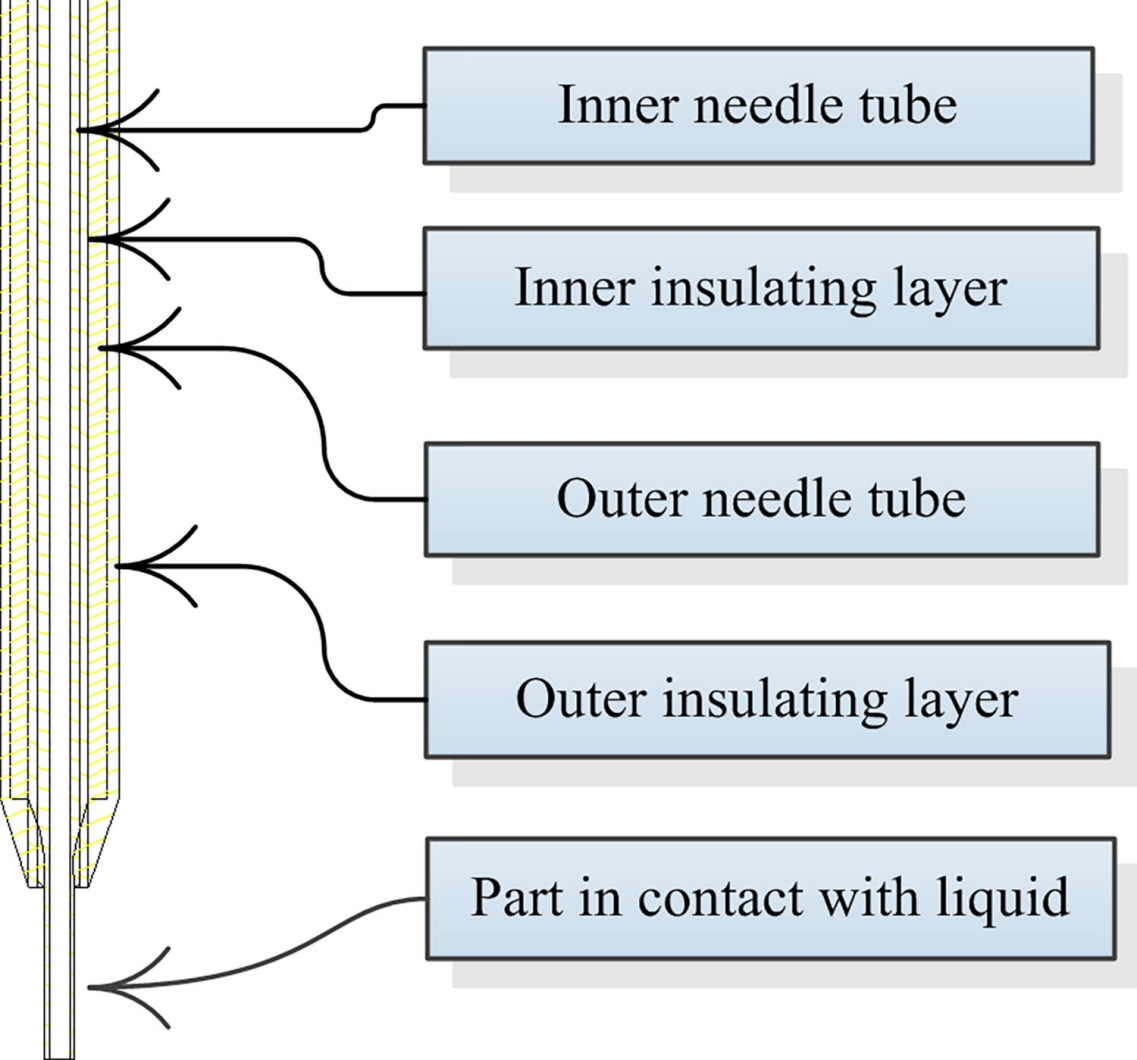
Appearance of probe.

The probe is an inner and outer coaxial needle, which is isolated with insulating material in the middle, and the appearance is shown in Fig 2. The inner needle tube and the outer needle tube are respectively connected to the liquid level detection circuit board with wires. The thinner part at the bottom of the needle is the part contacting the liquid. Generally, it is required not to insert the liquid level too deep (the insertion depth is about 2mm) to avoid carryover contamination and affect the measurement results [10].

### 2.2 Liquid level detection circuit board

The main function of the liquid level detection circuit board is to ensure that the probe can accurately detect the liquid level in the process of absorbing reagent, serum or other samples and discharging samples. Furthermore the probe cannot be inserted too deep into the liquid level. Secondly, it is necessary to prevent the probe from "empty suction" when the reagent and sample are insufficient [11]. If there is no liquid in the sample or reagent tube, or there is insufficient liquid to identify the liquid level, it will cause collision and bending of the probe. In addition, during the vertical movement of the probe, it may accidentally collide with other hard substances or the human body, causing damage to the probe or injury to the human body. So the liquid level detection circuit board also needs to have the function of anti-collision detection.

The liquid level detection signal and collision detection signal are both output to the probe drive board to control the movement of the probe.

**Fig 3:**
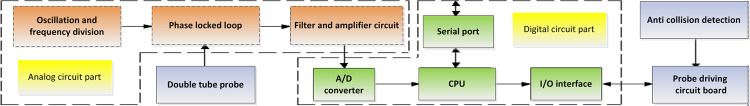
Structural diagram of liquid level detection circuit board.

The structure of liquid level detection circuit board is shown in Fig 3 below.

In the figure above, the part in the dotted line box is the main structure of the liquid level detection circuit board, which is divided into analog part and digital part. The analog part takes the PLL as the center. The front end is the oscillation and frequency division circuit to generate the reference signal. The double tube probe is connected to the circuit as a part of the PLL so that the probe capacitance of double tube probe becomes a part of the resonant capacitance. The back end of the analog part is connected with the signal processing circuit to filter and amplify the output signal by the PLL and finally output the processed analog signal to the digital circuit part. The digital part takes the CPU as the center and converts the analog signal into digital liquid level signal. Then the CPU analyzes and judges the signal and outputs the liquid level detection signal. Finally the detection signal is output to the probe driving circuit board through the input and output interface. In addition, the serial port can be designed to communicate with the host computer. As a relatively independent part of the liquid level detection circuit board, the collision detection circuit outputs the collision detection signal to the probe driving circuit board.

## 3 Key technologies and implementation

### 3.1 Design and implementation of the analog circuit

#### 3.1.1 Oscillation and frequency division circuit

**Fig 4:**
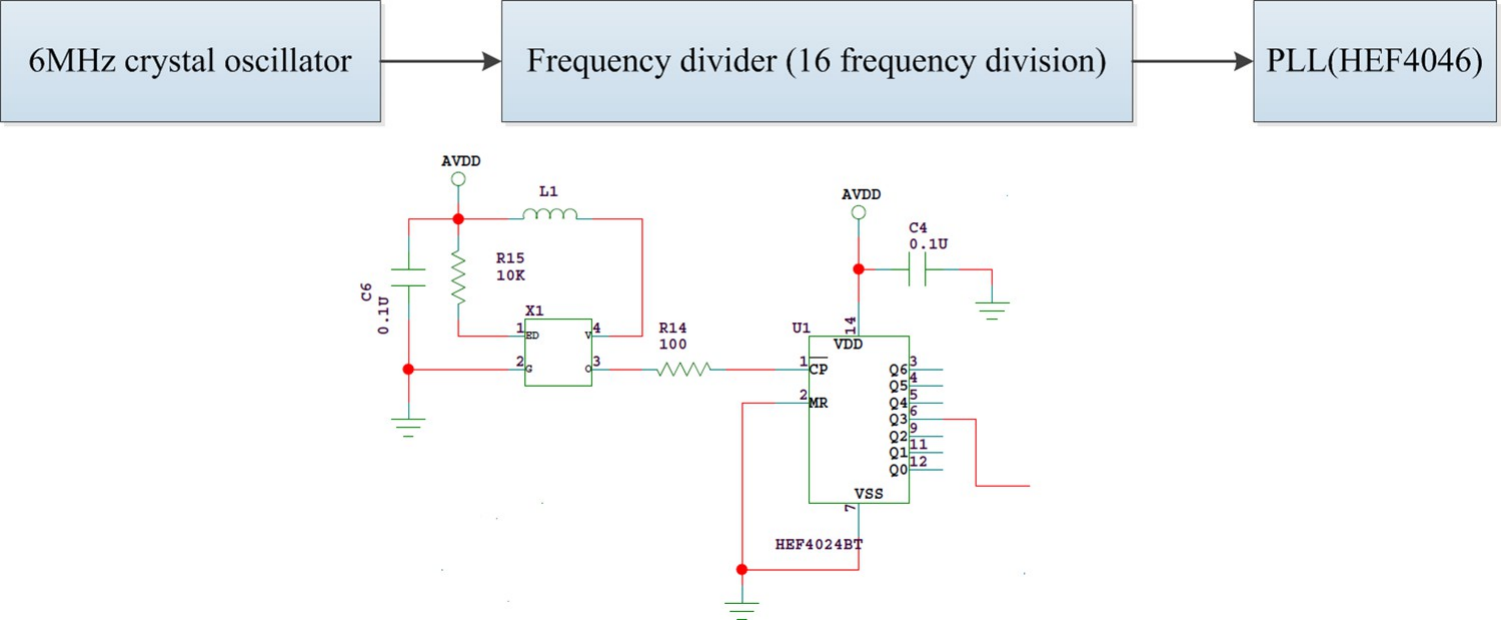
Oscillation and frequency division circuit.

The circuit includes a 6.0MHz crystal oscillator (X1) and a counter as shown in Fig 4. HEF4024 is selected as the counter and it can provide 2−2^7^ frequency division. The crystal oscillator output signal is used as the clock input of the counter. After 16 frequency division, the square wave signal with a frequency of 375 kHz is output as the reference input signal of the PLL chip HEF4046.

#### 3.1.2 PLL

**Fig 5:**
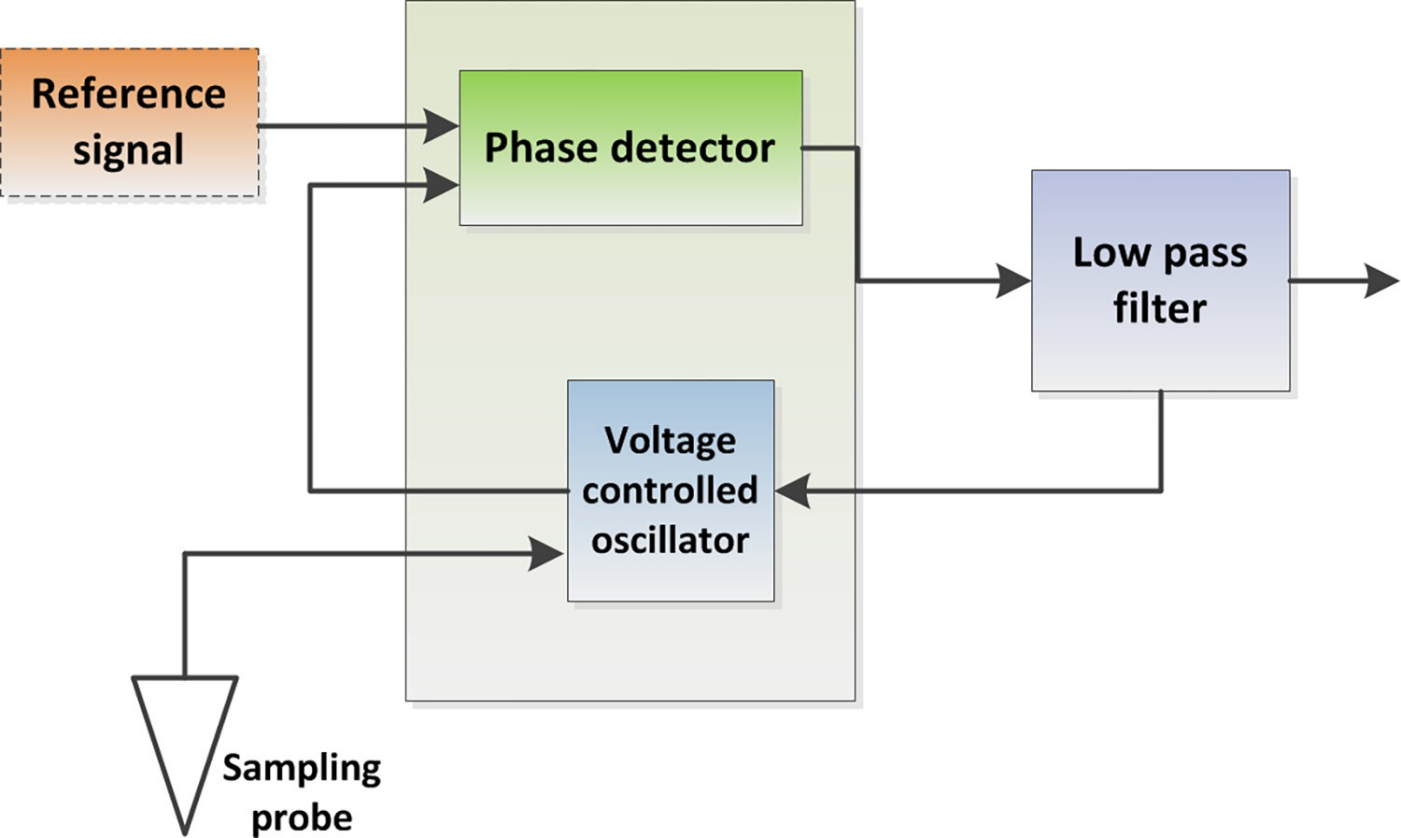
Structure diagram of PLL.

**Fig 6:**
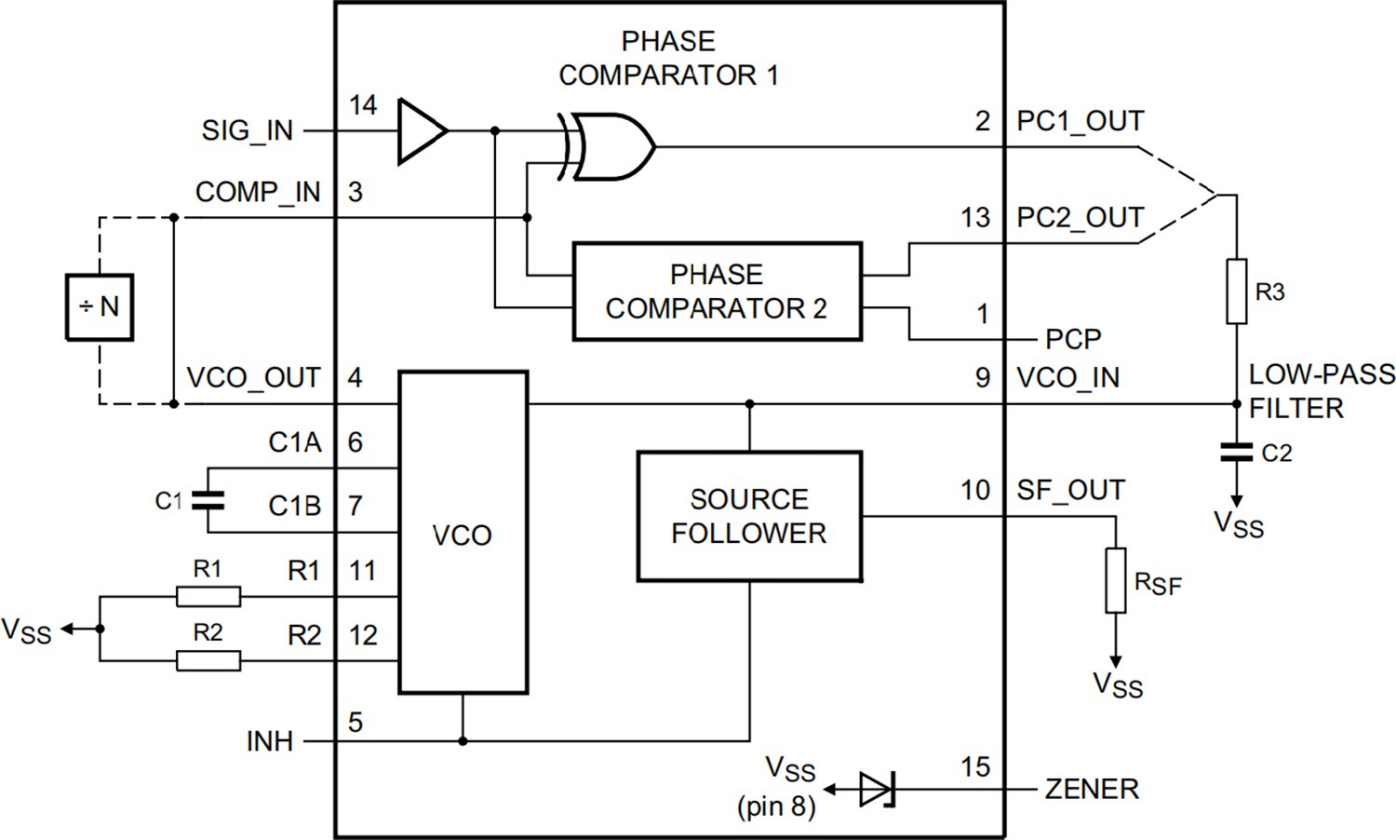
Functional diagram of PLL chip 4046.

Taking the PLL chip 4046 as the core, the double tube probe is connected to the circuit as part of the VCO tuning capacitor to control the output signal frequency of the VCO. The frequency divider signal and VCO output signal are input to the phase detector at the same time to get the error signal. Then the error signal passes through the low-pass filter to obtain the PLL output signal as shown in Figs 5 and 6. The signal is divided into two channels. One channel is added to the input of VCO to adjust the oscillation frequency so that the frequency of VCO output signal quickly approaches the frequency of reference signal to achieve phase locking. The other channel is output to the filter amplification circuit unit [12].

HEF4046BT is adopted as the PLL integrated circuit and its main working indexes are as follows. Working voltage: 5-15v; Operating frequency: About 2MHz at 10V; Temperature frequency stability: 0.04–0.05%/°C at 10V. The central frequency of VCO should not be too large. Through experimental test, when the central frequency is about 400kHz and the working voltage is 9V, good detection effect is achieved. Since the oscillation circuit adopts 6.0MHz crystal oscillator, the output frequency is 375 kHz after 16 frequency division, which meets the requirements. Considering the frequency offset caused by device tolerance, temperature drift and the change of tuning capacitance, it is necessary to determine the frequency offset of VCO according to experiments

Assuming a temperature change of 25°C, the frequency shifts Δf=0.05%×375kHz×25=4.69kHz. According to the previous experimental research on the capacitance and its variation rules of the liquid level detection probe, the capacitance increases when the probe contacts the liquid level, and the output voltage of the PLL also increases; When the probe leaves the liquid level, the capacitance gradually recovers, and the output voltage of the PLL also gradually recovers. In addition, when the probe contacts the liquid level, the change of the probe capacitance does not change with the change of the ambient temperature, but the probe capacitance will decrease with the decrease of the ambient temperature. According to the experimental data, the change rate is about 0.03pF/°C, equivalent to 0.12V/°C. The capacitance change of the probe is generally less than 3pF. If the upper limit is set as 3pF, the frequency offset is about 12kHz. Therefore the frequency offset is determined as f _L_ = 30kHz.

**Fig 7:**
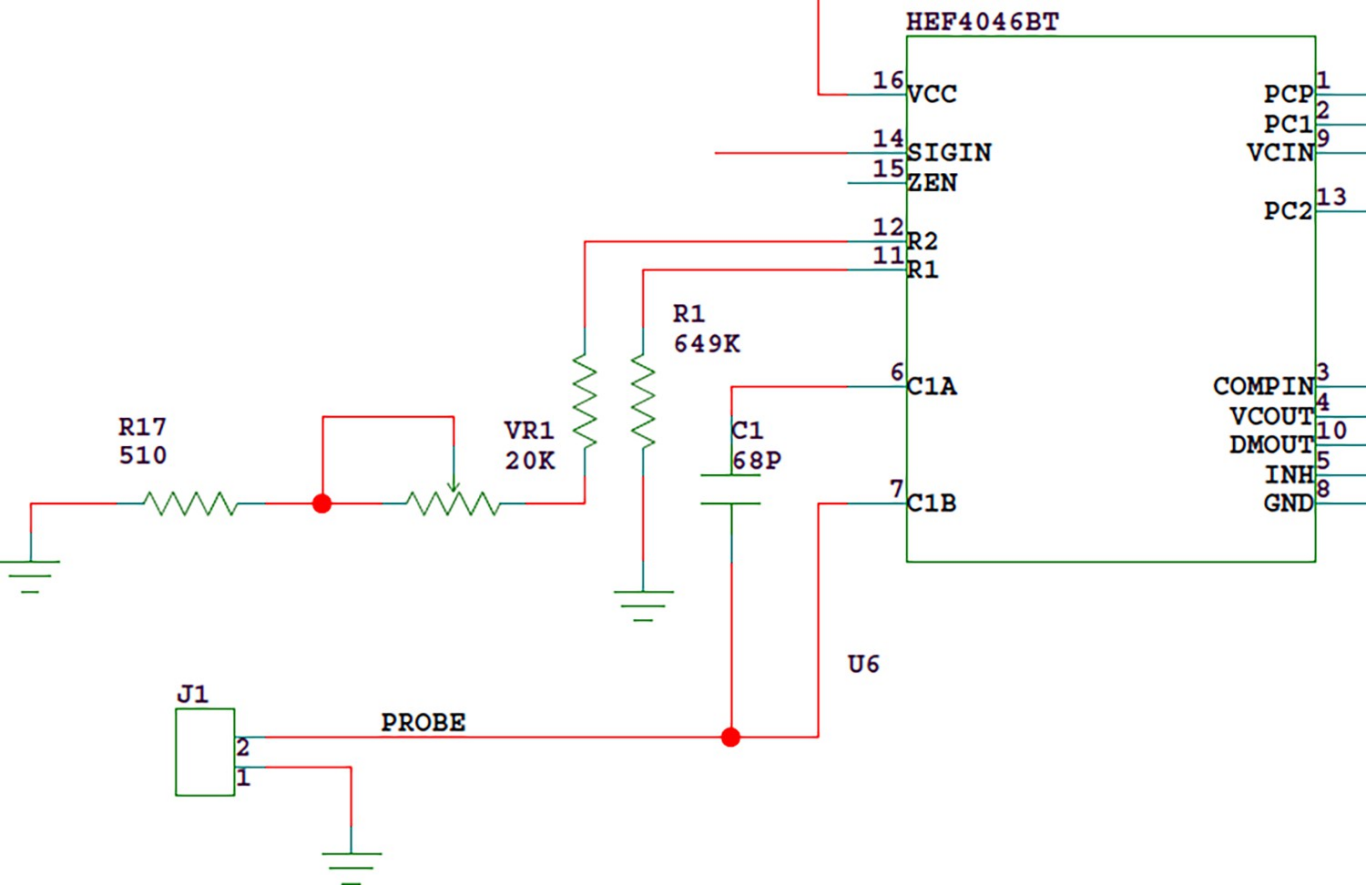
Connection mode between probe electrode and PLL.

In order to obtain high liquid level detection sensitivity, we can select a small capacitance value for C1 of VCO in Fig 6. If the working voltage of the circuit is set to 9V, C1 = 68pF can be selected. Since the inner and outer electrodes of the double tube probe are connected to the VCO tuning capacitor input of the PLL HEF4046 through connector J1, as shown in Fig 7, the actual VCO capacitance should also be added with the capacitance of the probe(about 24pF), so the capacitance between C1A and C1B is about 92pF.

**Fig 8:**
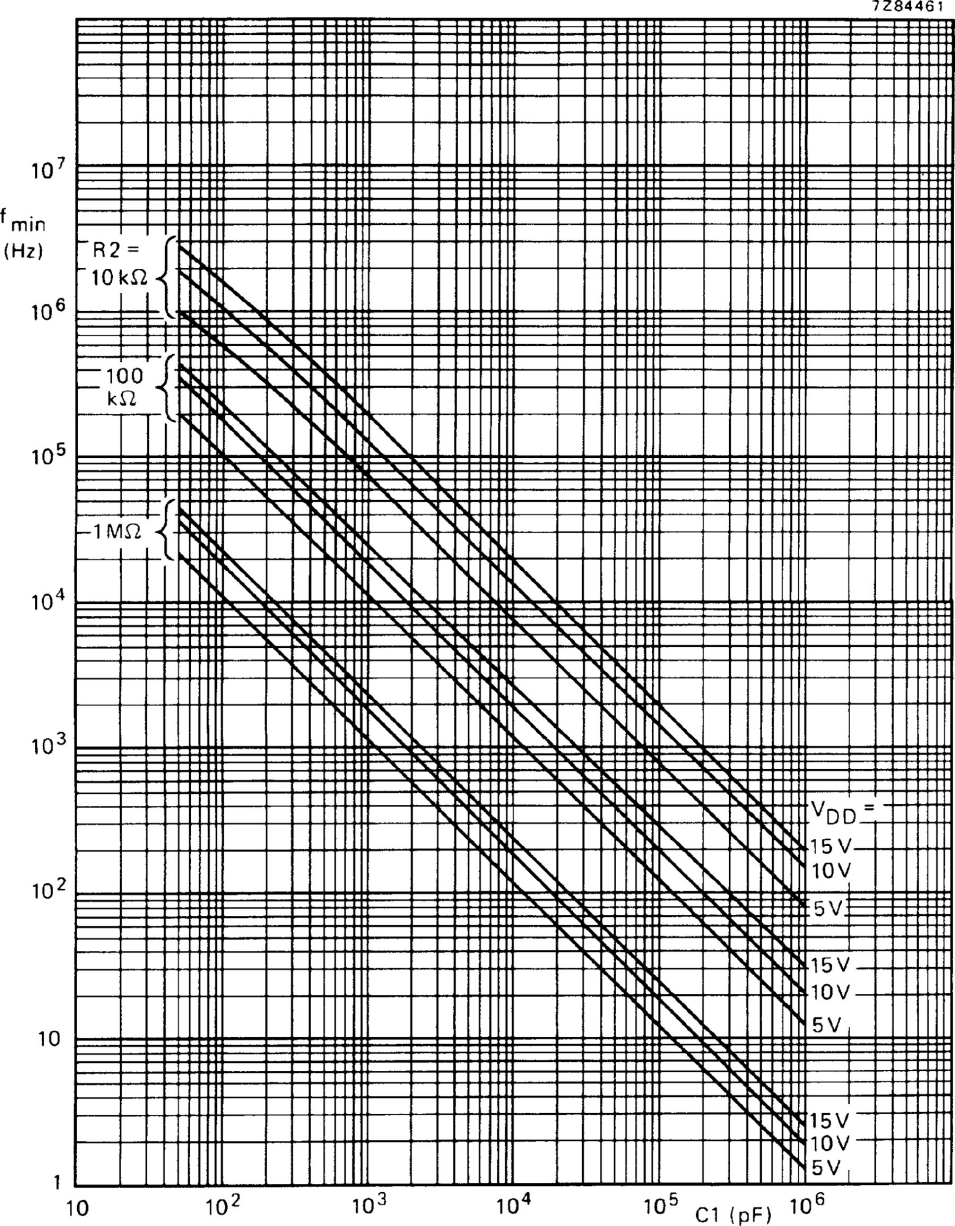
Frequency compensation curve of R2.

**Fig 9:**
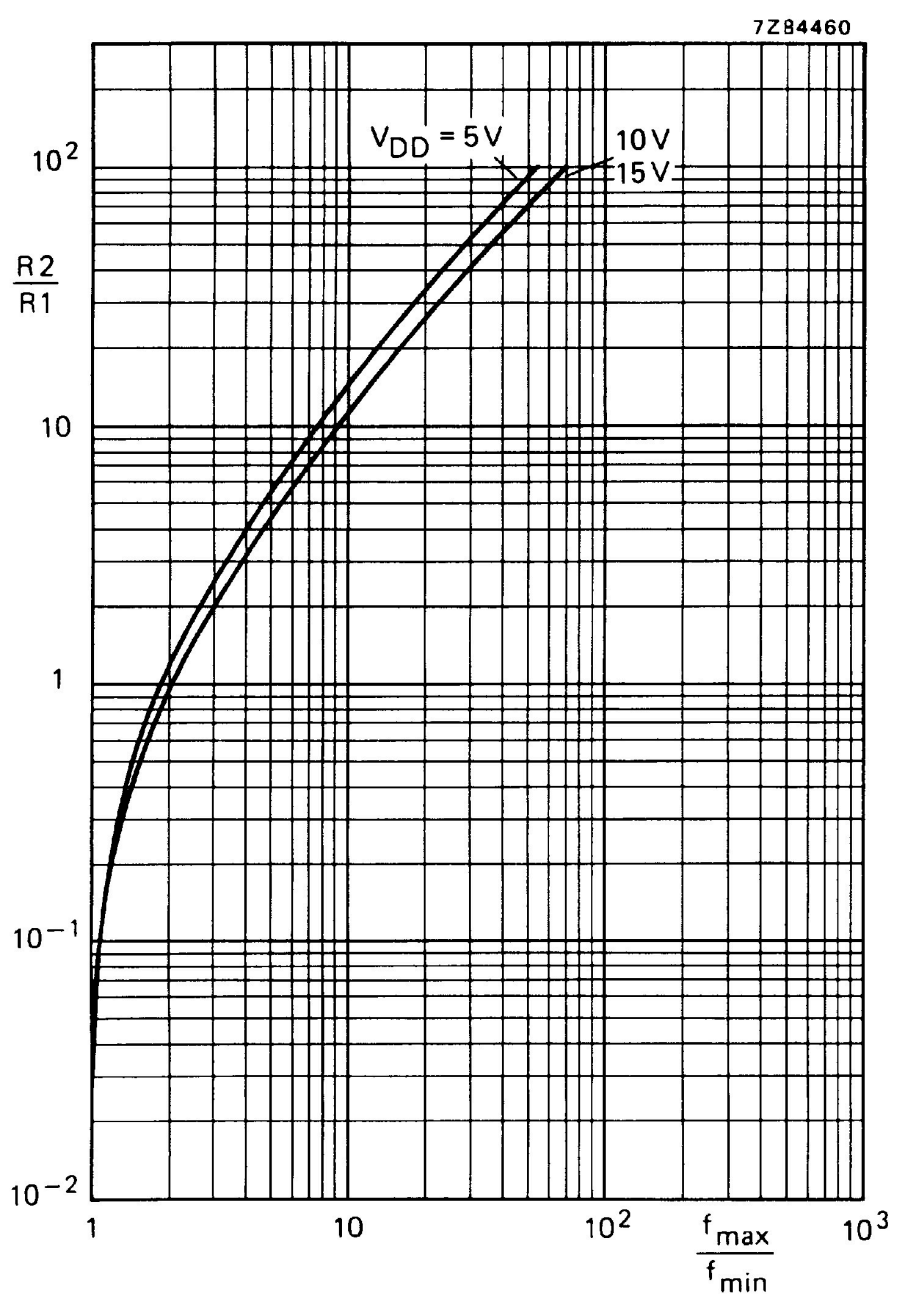
Relationship curve between R2/R1 and f_max_/f_min_.

The values of R2 and R1 in Fig 6 can be determined according to the characteristics of HEF4046BT as shown in Figs 8 and 9. First we determine R2. Set the center frequency f_O_ = 375kHz and the frequency offset $f_{L}=30\mathrm{kHz}$. Therefore, $f_{\min}{=f}_{O}{-f}_{L}=345\mathrm{kHz}$. C1 is about 92 pF. According to the frequency compensation curve 8 of R2, the value of R2 is about 57KΩ. In the actual circuit design process, R2 includes a fixed resistance of 43.2kΩ, an adjustable resistance of 20KΩ and a toggle switch to facilitate adjustment.


$$\left(T_{amb}=25{}^{\circ}C;VCO_{IN}\;at\;V_{ss};INH\;at\;V_{ss};R1=\infty \right)$$

Next, determine R1.According to f_O_ = 375kHz, f_L_ = 30kHz, fmaxfmin=fO+fLfO−fL=405345=1.17. According to the relationship curve between R2/R1 and f_max_ /f_min_, R1=57KΩ0.1=570KΩ.

**Fig 10:**
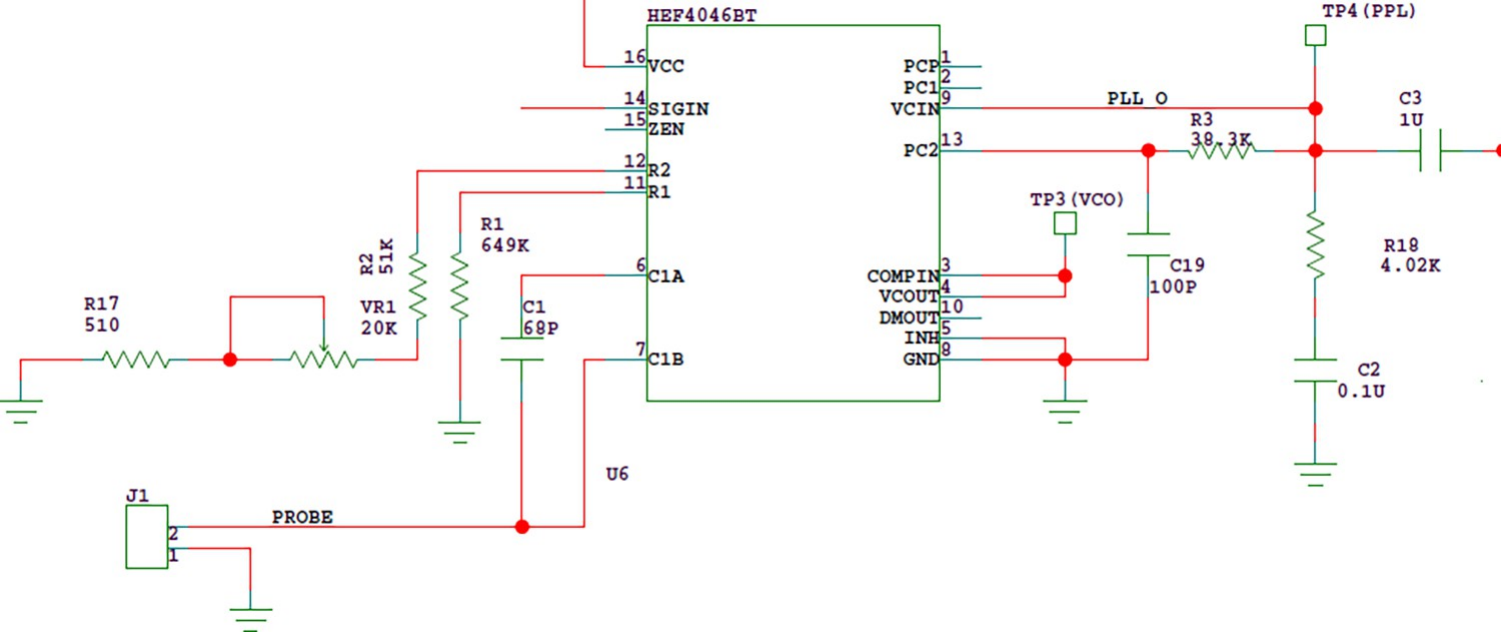
Adjusting circuit of PLL operating point.

In order to make the PLL chip lock the set frequency range normally, the control voltage is required to reach an appropriate range. The working voltage of HEF4046BT is set to 9V, and the control voltage VCIN(the voltage of test point TP4 in Fig 10) is required to reach the range of 0.8V-8.3V. Considering from the following two aspects, VCIN is set to 3.5V at room temperature:①when the probe contacts the liquid level, VCIN will rise, so in order to ensure a large dynamic range, VCIN should be as low as possible;②As the ambient temperature decreases, the probe capacitance decreases and the VCIN decreases. According to the experimental data, the change rate is about 0.12V/°C, that is, when the temperature decreases from 25°C to 5°C, VCIN decreases by about 2.4V. In order to ensure that the liquid level detection can work normally within the temperature range of 5°C ~ 35°C, VCIN can not be set too low.

**Fig 11:**
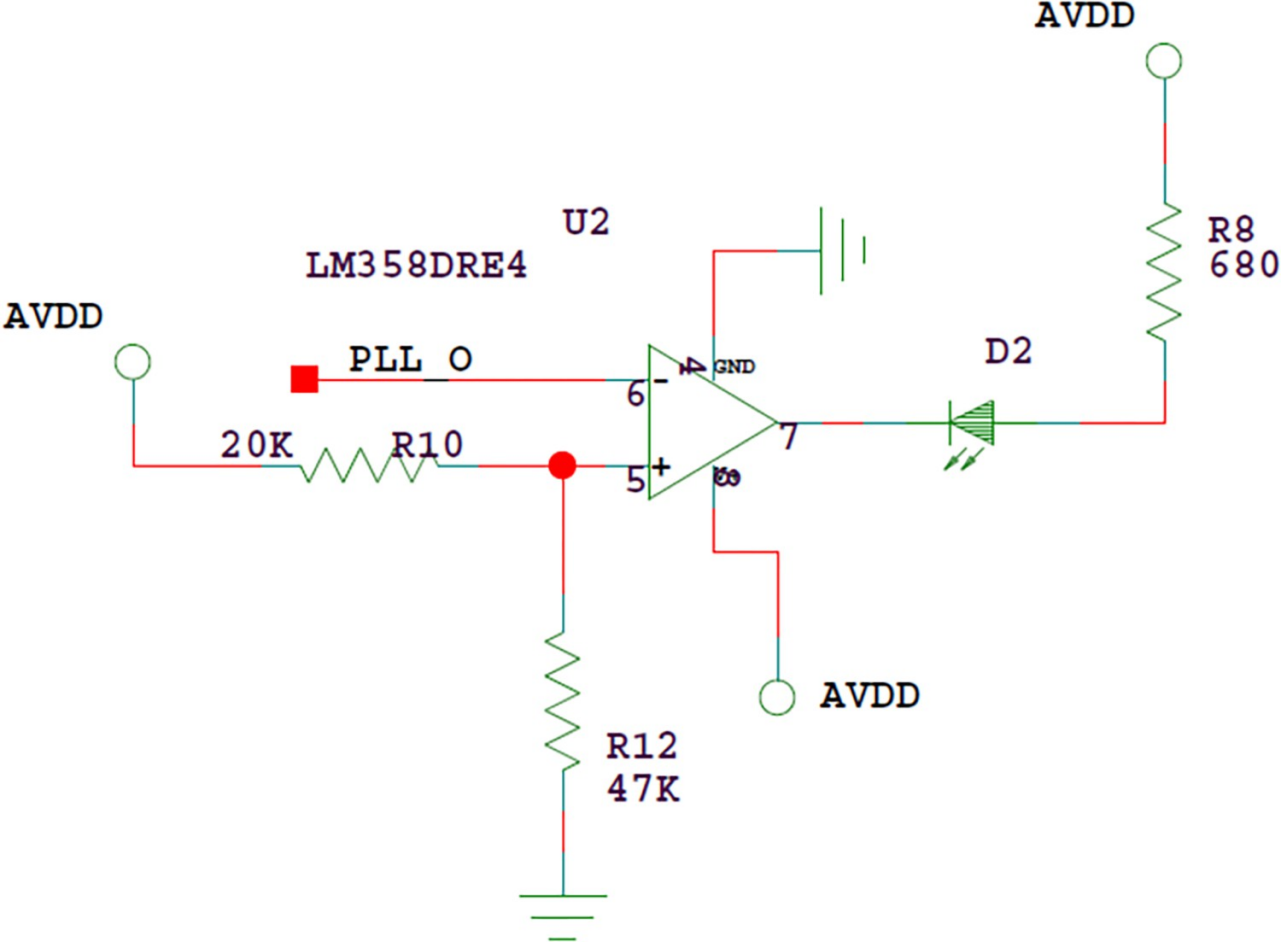
LED display circuit.

Adjust the variable resistor VR1 to make the TP4 voltage reach 3.5V, and set the LED display circuit, as shown in Fig 11.The voltage divider is composed of resistors R10 and R12, so that the in-phase input voltage of U2 is 3.5V. Adjust VR1. When the TP4 voltage exceeds 3.5V, that is, the inverted input voltage of U2 is higher than the in-phase input voltage, the LED indicator D2 is on, otherwise D2 is off.

**Fig 12:**
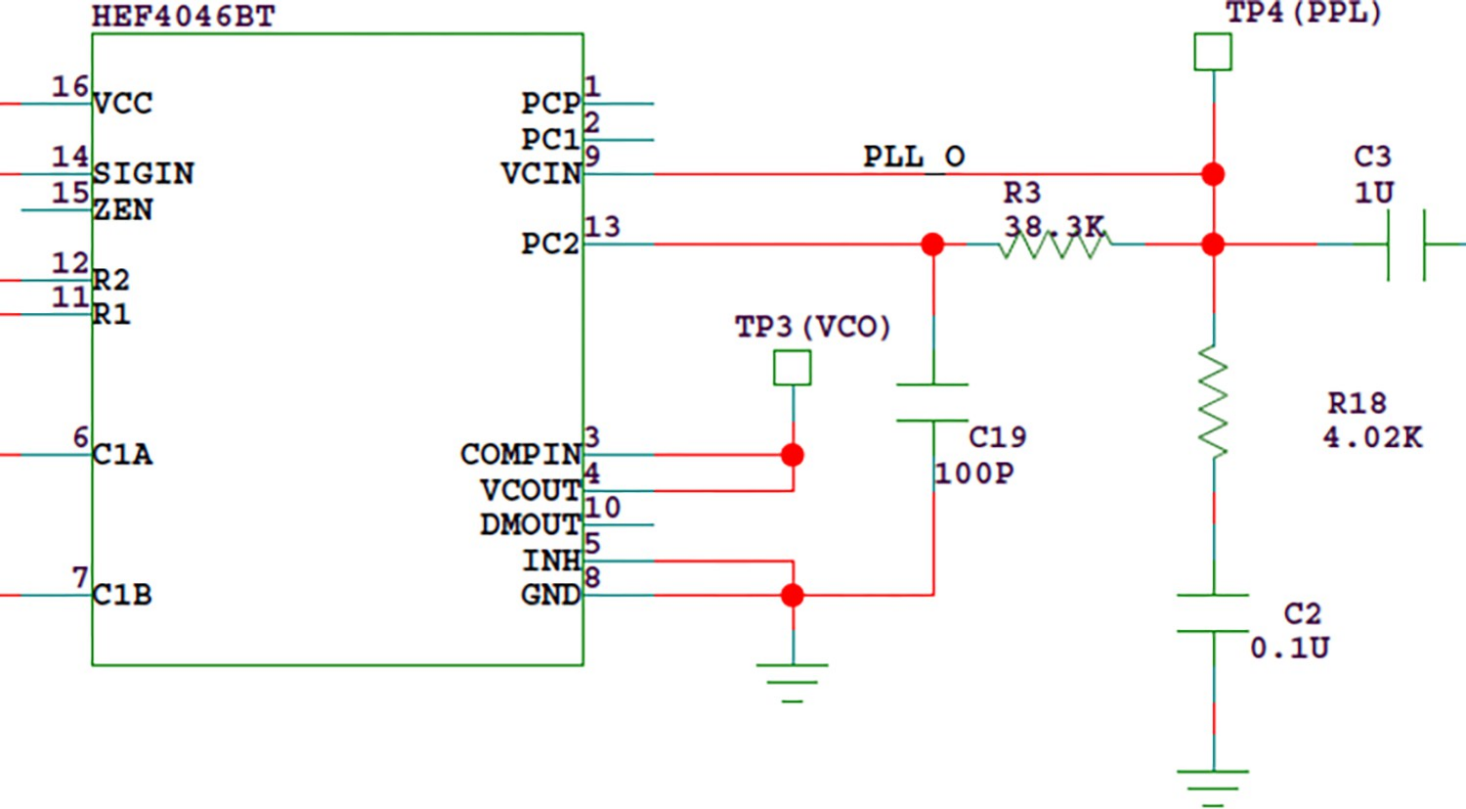
Proportional integral filter.

A low-pass filter shall be connected after the control voltage VCIN, as shown in Fig 12. The low-pass filter is a proportional integral filter composed of resistors R3, R18 and capacitor C2. Although the proportional integral filter has poor suppression of high-frequency noise, it can improve the system stability, fast capture performance, filtering performance and traction capacity, so it is widely used in PLL.

The transfer function of the passive proportional integral filter is:

$$\mathrm{KF}\;(s)=\frac{1+S\tau_{2}}{1+S(\tau_{1}+\tau_{2})}$$ (1)

In the above formula, $\tau_{1}{=R}_{3}C_{2}$, $\tau_{2}{=R}_{18}C_{2}$. According to references [13, 14], combined with the application scenario of the system, the natural angular frequency and damping coefficient of the PLL can be obtained by calculation: ω_n_ = 2.311*10^3^rad/s (f_n_ = 368Hz); ξ = 0.515。

#### 3.1.3 Signal processing circuit

**Fig 13:**
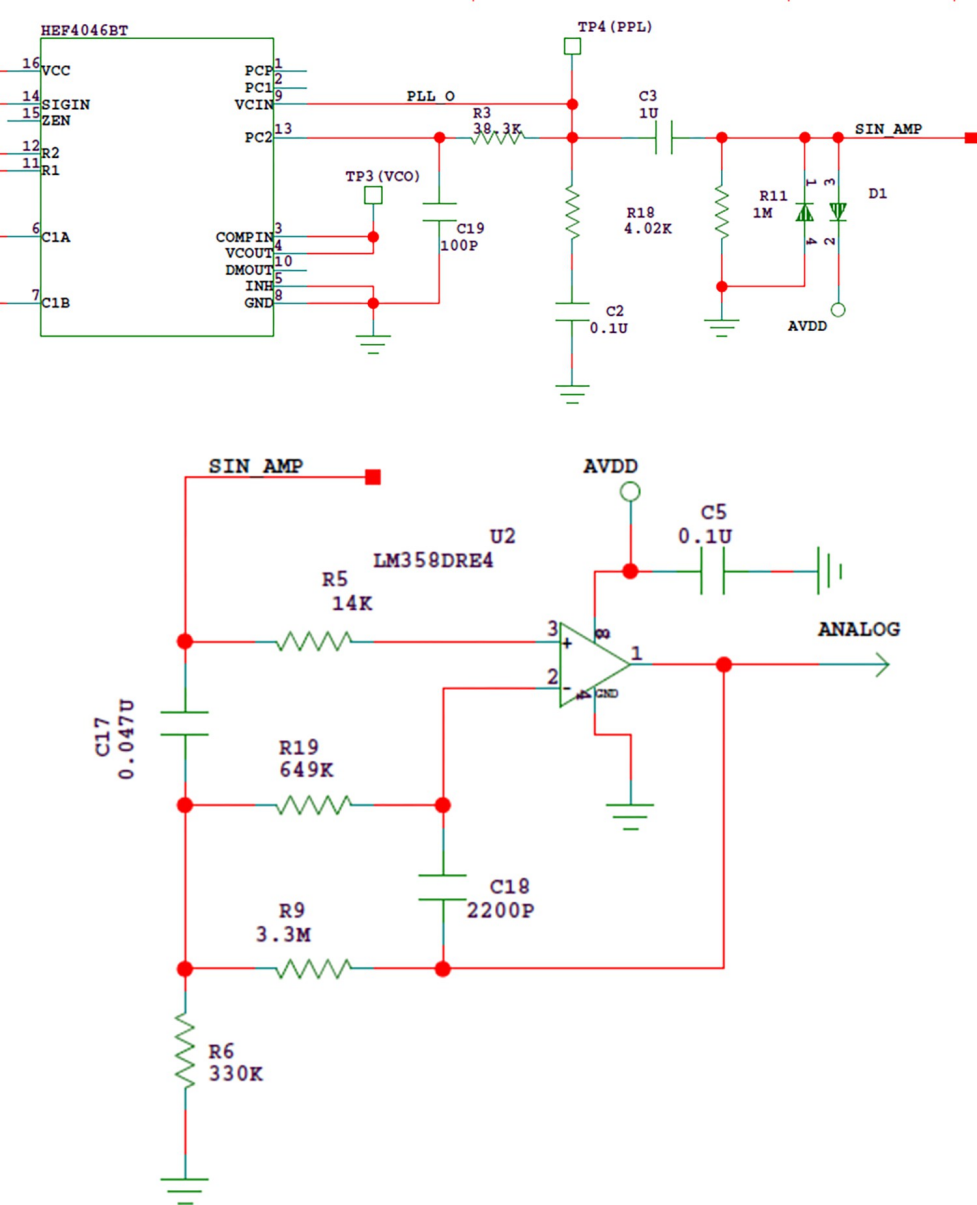
(a) Signal processing circuit. (b) Signal processing circuit.

The main function of the signal processing circuit is to filter and amplify the signal output by the PLL, let the signal in the passband pass through, suppress the out-of-band interference, amplify the signal and improve the signal-to-noise ratio. The circuit diagram is as shown in Fig 13(A) and 13(B).

In Fig 13(A), the output signal of the proportional integral filter composed of R3, R18 and C2 first passes through the first-order passive high pass filter composed of C3 and R11, which mainly plays the role of DC isolator. The fundamental frequency of the signal is 0.1Hz, thus the parameters of the high pass filter are designed as C_3_ = 1uF,R_11_ = 1MΩ,then: $\tau =R_{11}C_{3}=1S$, fn=R11C32π=0.16Hz.

In order to filter the interference and amplify the signal amplitude at the same time, an active multi-channel feedback low-pass filter is designed, as shown in Fig 13(B). Operational amplifier LM358 is selected as the core device. According to the experimental test results, the amplitude of the liquid level detection signal output by the high pass filter is about 0~0.4V. When the probe does not contact the liquid level, it is about 0, and when it contacts the liquid level, it is 0.3V~0.4V. Therefore, as long as the liquid level detection signal is amplified by 10 times, it can meet the processing requirements of subsequent circuits. So, the gain H_0_ of the active low-pass filter is designed as 10. Since the fundamental frequency of the signal is 0.1Hz, the tenth harmonic is 1Hz, and the cut-off frequency f_c_ of the low-pass filter can be designed as 10 times of the tenth harmonic of the signal. Therefore, the cut-off frequency f_c_ of the low-pass filter is designed as 10Hz.

The corresponding indexes are: gain H_0_ = 10, damping coefficient ξ = 0.772, characteristic frequency f_n_ = 10.7hz. Relevant calculations are as follows:

H0=R9R6=3.3M330K=10 (2)

$$\xi =\frac{1}{2}\sqrt{\frac{C_{18}}{C_{17}}}(\sqrt{\frac{R_{19}}{R_{9}}}+\sqrt{\frac{R_{9}}{R_{19}}}+\frac{\sqrt{R_{9}R_{19}}}{R_{6}})=0.772$$ (3)

$$f_{n}=\omega_{n}/2\pi =\sqrt{\frac{1}{R_{9}R_{19}C_{17}C_{18}}}/2\pi =10.7\mathrm{Hz}$$ (4)

The low-pass filter amplifies the useful signal by 10 times and suppresses the interference signal by 50%. The original signal-to-noise ratio is 1:1, after filtering, the signal-to-noise ratio can be increased to 20:1.

### 3.2 Design and implementation of digital circuit

**Fig 14:**
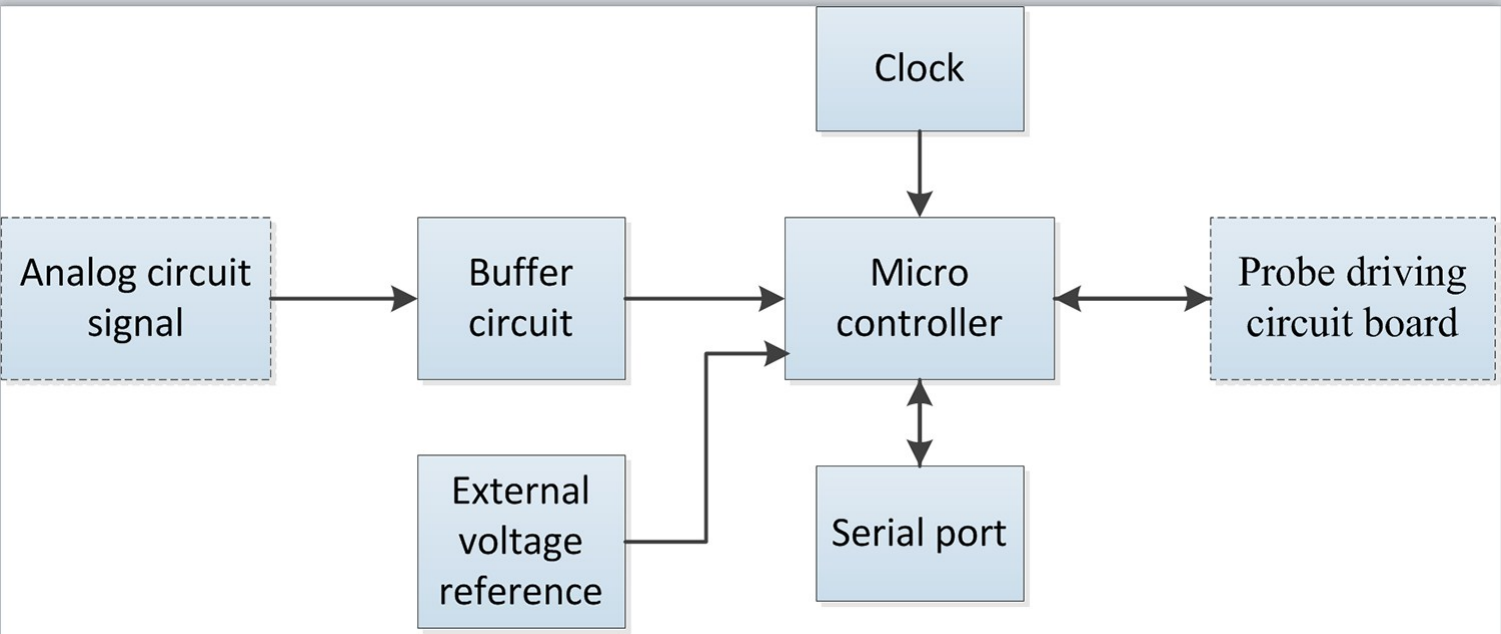
Diagram of digital circuit.

The main functions of the digital circuit include: A / D conversion of the analog signal of liquid level detection; outputing liquid level detection signal; providing clock circuit, reset circuit and level reference circuit for microcontroller; providing signal indicator; connecting to the computer through the communication interface and supporting online download, as shown in Fig 14.

**Fig 15:**
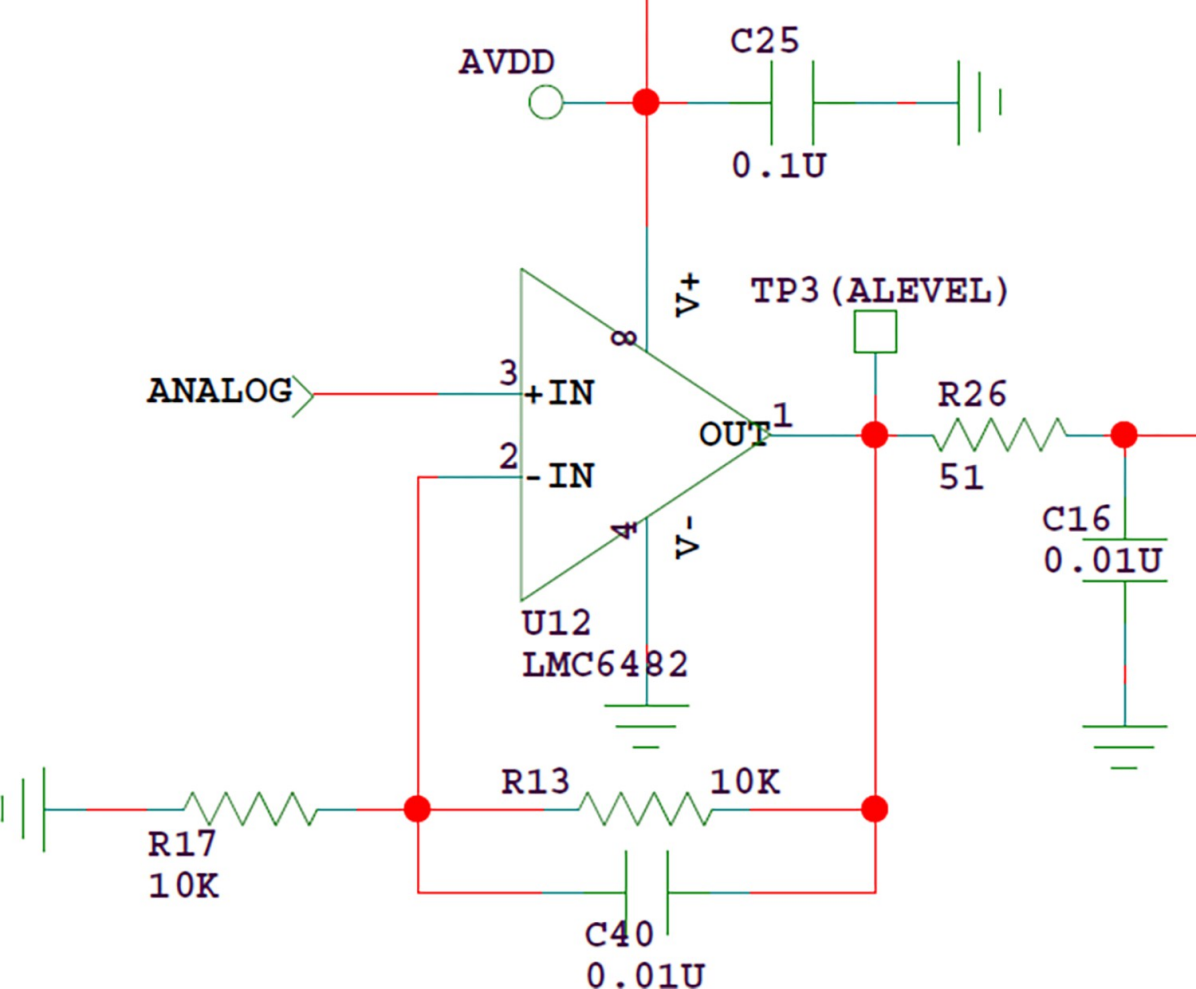
A / D input buffer circuit.

The buffer circuit is a voltage follower. The signal ANALOG in Fig 15 is the analog output signal in Fig 13(B) and the output resistance is very small. The main purpose of R26 and C16 is not low-pass filtering, but to avoid the sudden change of ADC input level and protect the microcontroller. After the liquid level detection signal is input into the buffer circuit, it is output to the microcontroller for A/D conversion.

When judging the liquid level signal, the combination of slope detection and amplitude detection is used. Before the sampling probe contacts the liquid, the analog signal voltage of liquid level detection is very small (almost 0V). When the probe contacts the liquid level, the analog signal voltage will rise. But when detecting trace sample liquid, the amplitude of the analog signal voltage changes gently. If only the amplitude threshold detection method is used at this time, false detection may occur. Therefore, the signal slope detection method is more suitable in this case. The specific judgment method is to set two amplitude thresholds. When the amplitude of the liquid level analog signal exceeds the high threshold, it is judged that the probe contacts the liquid level; When it exceeds the low threshold but does not reach the high threshold, the signal change slope is detected. When the slope meets the detection conditions, it is judged that the probe contacts the liquid level.

### 3.3 Anti-collision detection circuit and interface

**Fig 16:**
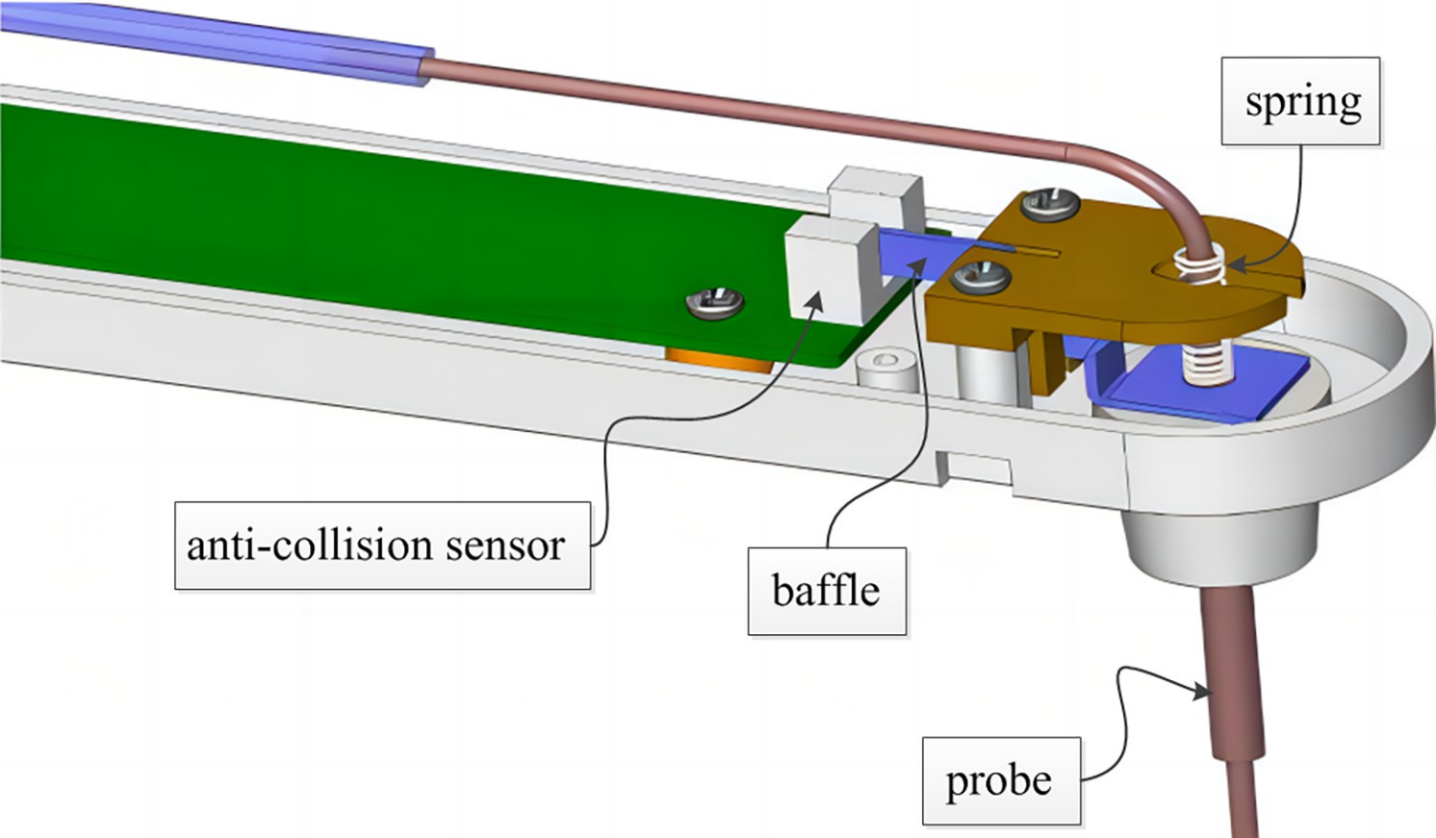
Schematic diagram of vertical anti-collision structure.

**Fig 17:**
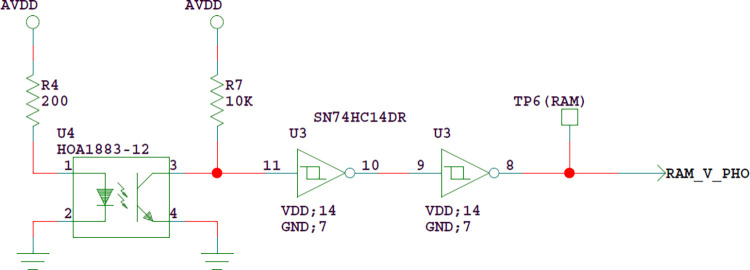
Anti-collision detection circuit.

The anti-collision detection circuit mainly detects whether the probe has an accidental impact during movement, and protects the double tube probe while ensuring personal safety. The core component of anti-collision detection circuit is anti-collision sensor HOA1883-12, which outputs pulse signal to microcontroller. When the probe moves normally, the baffle is between the LED and the photoelectric triode, and the sensor output is high level; In case of accidental impact of the probe, the baffle cannot cover the LED, and the sensor output becomes low, as shown in Figs 16 and 17.

The anti-collision detection signal is driven by SN74HC14, connected to the filter capacitor and output to the probe driving circuit board to control the movement of the probe.

The power supply of the whole system is provided by the probe drive circuit board with +12V power supply. When the probe does not touch the liquid level, the liquid level detection circuit board outputs a low level to the probe driving circuit board; Once the probe contacts the liquid level, it outputs a high level.

### 3.4 Reliability and electromagnetic compatibility (EMC) design

The reliability and EMC of the whole system are mainly considered from the following aspects [15].

#### 3.4.1 Filtering decoupling processing of input power supply and power supply terminal of each IC (including crystal oscillator)

The input interface includes GND and +12V power supply, which is connected close to the socket and connected to two filter capacitors(10Μf and 0.1Μf). The +5V and +9V power supplies are obtained from the +12V power supply through DC-DC conversion. Among them, analog +5V power supply and digital +5V power supply are isolated by magnetic beads. The output terminals of +5V and +9V power supplies are connected with filter capacitors. The VCC of each IC chip, including the VDD of the crystal oscillator, is terminated with 0.1Μf filter capacitor, and the capacitor is close to the corresponding pin.

#### 3.4.2 Separate layout of analog circuit and digital circuit

The analog signal is connected to the analog input of the micro controller through the emitter follower. The analog circuit and digital circuit are arranged separately, and the crystal oscillator is close to the clock input port of the micro controller. A filter inductor is added between the analog power supply and the digital power supply, and each power supply is connected with a terminal filter capacitor.

#### 3.4.3 Output interface

The crystal oscillator is located away from the liquid level detection signal interface. All signal interfaces are connected with matching resistors and filter capacitors.

## 4 Analysis of test results

The environmental conditions for the test are shown in Table 1 below.

**Table 1:** Test environmental conditions.

Item	Allowed band	Unit	Measured value
Ambient temperature	15~30	°C	22~27
Relative humidity	< 80%	/	50~75
Atmospheric pressure	101000 ± 4000	Pa	100000
Supply voltage	220 ± 10%	V	210~230
Power frequency	50 ± 1	Hz	50

### 4.1 Power supply voltage test

The test requirements are as follows: when the input power supply voltage is 12V within the range of 11.4v ~ 12.6V, the output of 5V generated by 78L05ACM and 5V generated by AD586KRZ is within the range of 4.75V ~ 5.25V, the output of 9V is within the range of 8.55V ~ 9.45v, and the ripple peak-to-peak voltage of power supply voltage is not greater than 100mV.

The test results are shown in Table 2 below.

**Table 2:** Test data of the power supply voltage and ripple.

Tested power supply /V	Input power/V	Output voltage/V	ripple peak-to-peak voltage/mV
5(Analog)	11.4	5.007	70
	12	5.008	60
	12.6	5.009	70
5(Digital)	11.4	4.998	73
	12	4.998	69
	12.6	4.999	74
9(Digital)	11.4	9.22	78
	12	9.21	71
	12.6	9.22	76

The output of two 5V power supply and 9V power supply are within the allowable range, and the ripple peak-to-peak voltage of each power supply is about 70mV. Within the allowable range, the test passes.

### 4.2 Signal quality test of 6MHz clock and frequency division circuit

Test requirements: the frequency of clock signal shall be 6MHz, and the deviation shall not exceed ± 1%. The signal shall be square wave and there shall be no spike or burr higher than 0.5V; The signal of the frequency division circuit shall be 375khz, and the deviation shall not exceed ± 1%.

**Fig 18:**
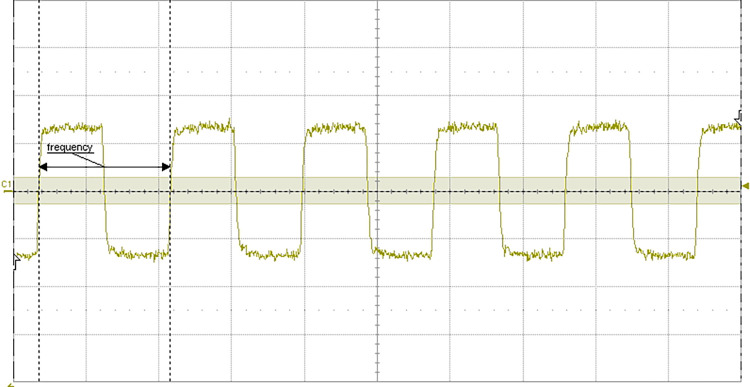
6MHz clock signal.

**Fig 19:**
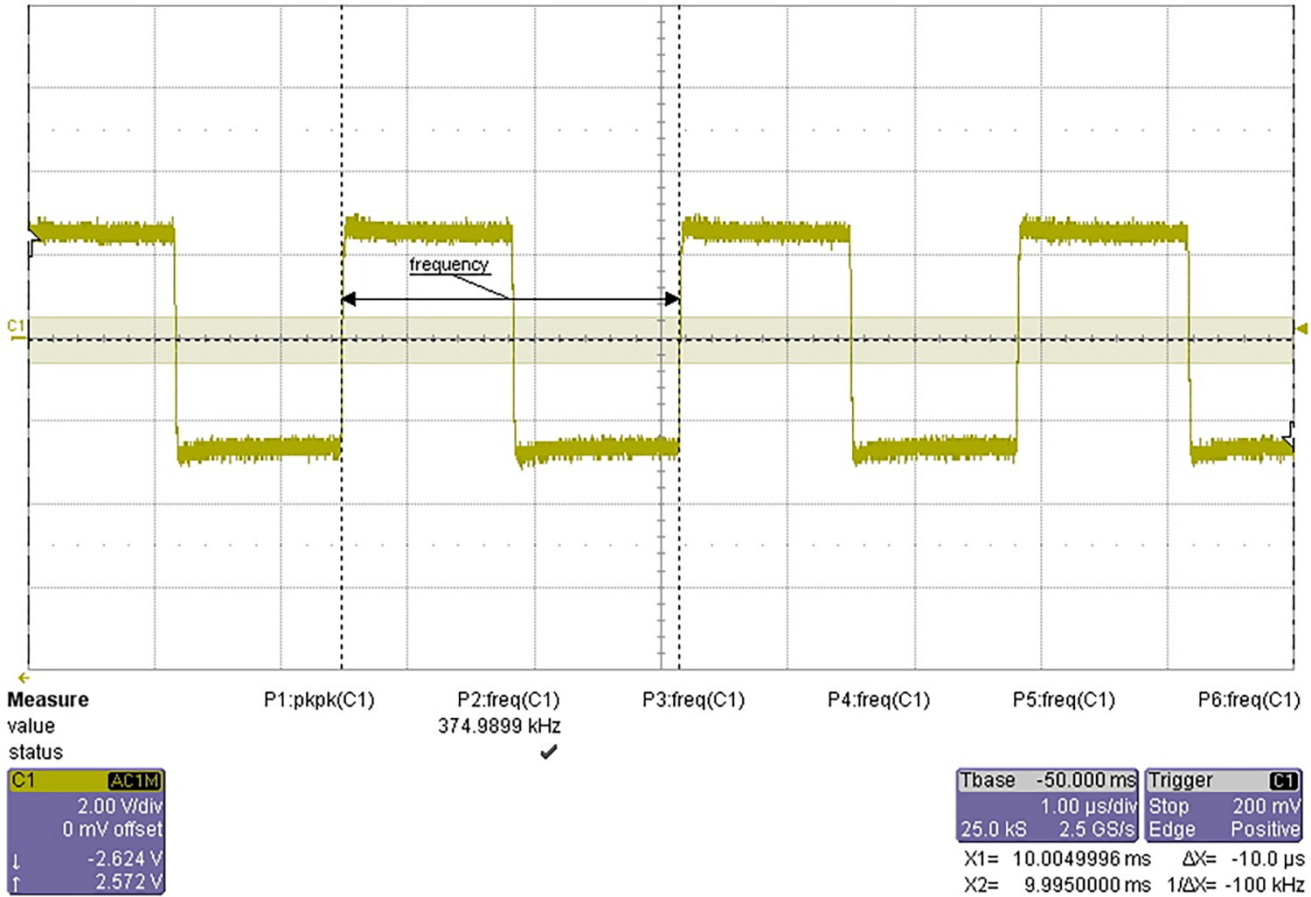
375 kHz square wave signal.

The test results are shown in Figs 18 and 19.

The frequency range of 6MHz clock signal is 5.99 MHz ~ 6.01 MHz, and the range of frequency division is 374.90kHz ~ 375.03kHz. The signal quality meets the design requirements and passes the test.

### 4.3 Measurement of central oscillation frequency and voltage control characteristics of PLL

#### 4.3.1 Test of central oscillation frequency

The test requirements are: the center frequency of VCO of the PLL shall be equal to the reference frequency: 375 kHz. According to the datasheet of HEF4046, the allowable frequency offset is 3.6kHz when the test ambient temperature is 24°C.

**Fig 20:**
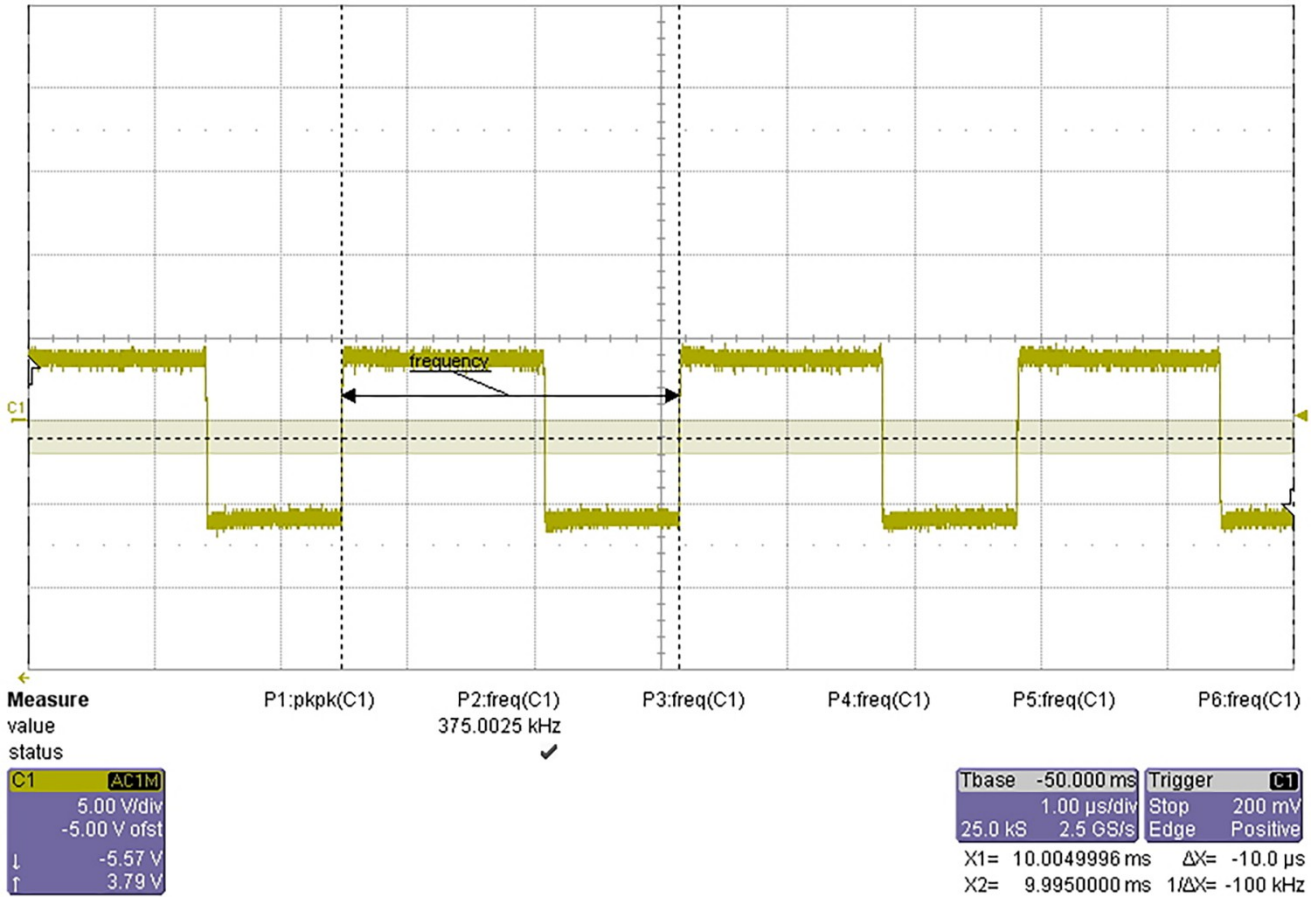
Output signal of VCO of the PLL.

The test results are shown in Fig 20.

The output signal quality of VCO meets the design requirements, and the central oscillation frequency range is 374.90kHz ~ 375.13kHz. The test is passed.

#### 4.3.2 Test of voltage control characteristic

The test requirements are: the PLL working parameters are designed reasonably. According to the characteristics of the chip, the VCIN should be in the range of 4.5 ± 2.5V, i.e. 2V ~ 7V, and the VCOUT linearity is 0.25%.

**Fig 21:**
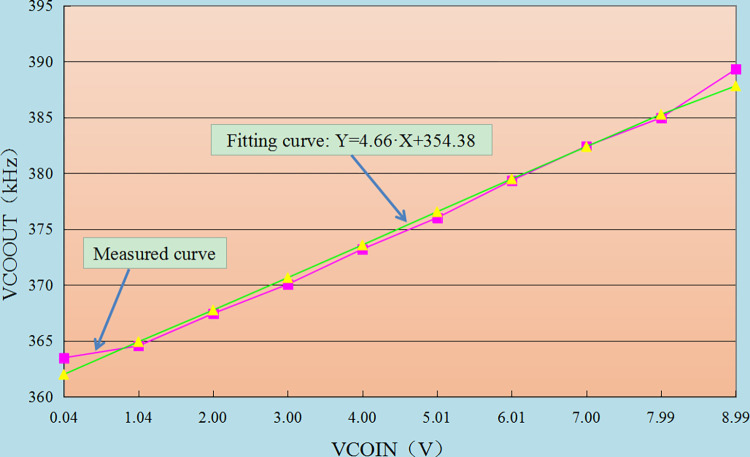
Characteristic curve of voltage controlled oscillator of PLL.

The test results are shown in Table 3 and Fig 21.

**Table 3:** Test results of voltage control characteristics.

VCOUT/V	Measured frequency/kHz	Corresponding frequency on regression line/kHz	Frequency deviation/kHz	Allowable frequency deviation /kHz
0.04	363.48	361.99	-1.49	3.63
1.04	364.55	364.92	0.37	3.66
2.00	367.44	367.75	0.31	3.69
3.00	370.06	370.65	0.59	3.72
4.00	373.21	373.58	0.37	3.75
5.01	376.01	376.55	0.54	3.78
6.01	379.35	379.49	0.14	3.81
7.00	382.42	382.38	-0.04	3.84
7.99	384.96	385.27	0.31	3.87
8.99	389.30	387.81	-1.49	3.91

When the frequency output of VCOUT is in the range of 2V~7V, it meets the requirement of linearity(0.25%). The design of PLL working parameters is reasonable, meets the design requirements, and passes the test.

### 4.4 Test of 11.0592MHz clock signal quality

Test requirements: the frequency of clock signal shall be 11.0592MHz and the deviation shall not exceed ±1%, i.e. 11.0592 ± 0.11 MHz. The signal shall be square wave and there shall be no spike or burr higher than 0.5V.

**Fig 22:**
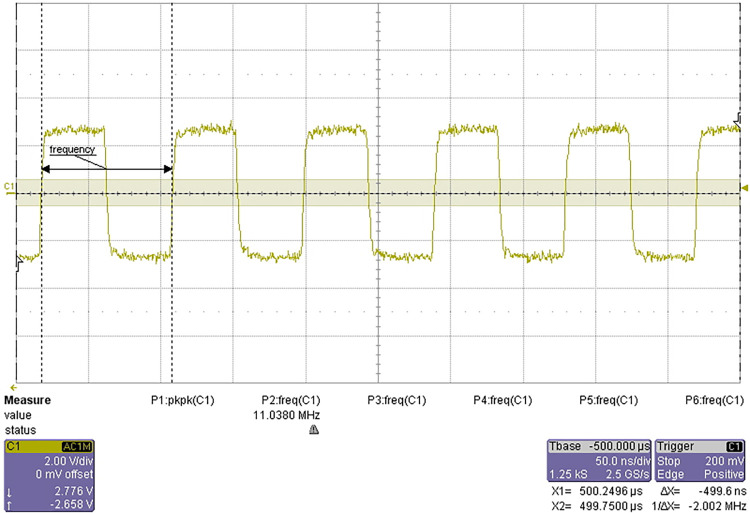
Waveform of 11.0592MHz clock signal.

The test results are shown in Fig 22.

The clock signal quality meets the design requirements, and the frequency range is about 11.0750MHz~11.0380MHz. The test is passed.

### 4.5 Test of reset function

The test requirements are: the reset signal can be output correctly, and the high level shall be maintained for at least 24 clock cycles.

**Fig 23:**
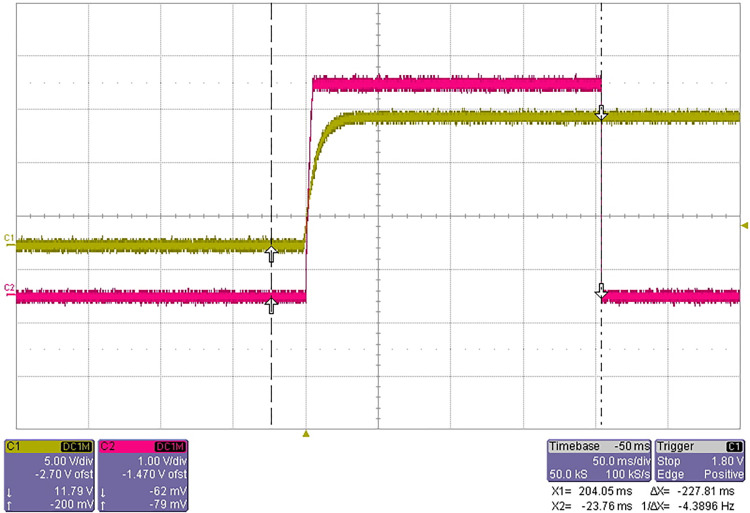
Reset signal waveform.

The test results are shown in Fig 23.

In Fig 23, the red waveform is the reset signal and the yellow waveform is the 12V power waveform. The reset signal can respond correctly, and the high level of the reset signal lasts about 200ms. The reset waveform has no burr, the signal quality meets the design requirements, and the test passes.

### 4.6 Test of liquid level detection function

The test requirements are: when the probe contacts the liquid level, the liquid level detection circuit board can timely and reliably output the liquid level detection signal. That is, when the probe does not contact the liquid level, the liquid level detection signal is low level, and when it contacts the liquid level, it becomes high level.

**Fig 24:**
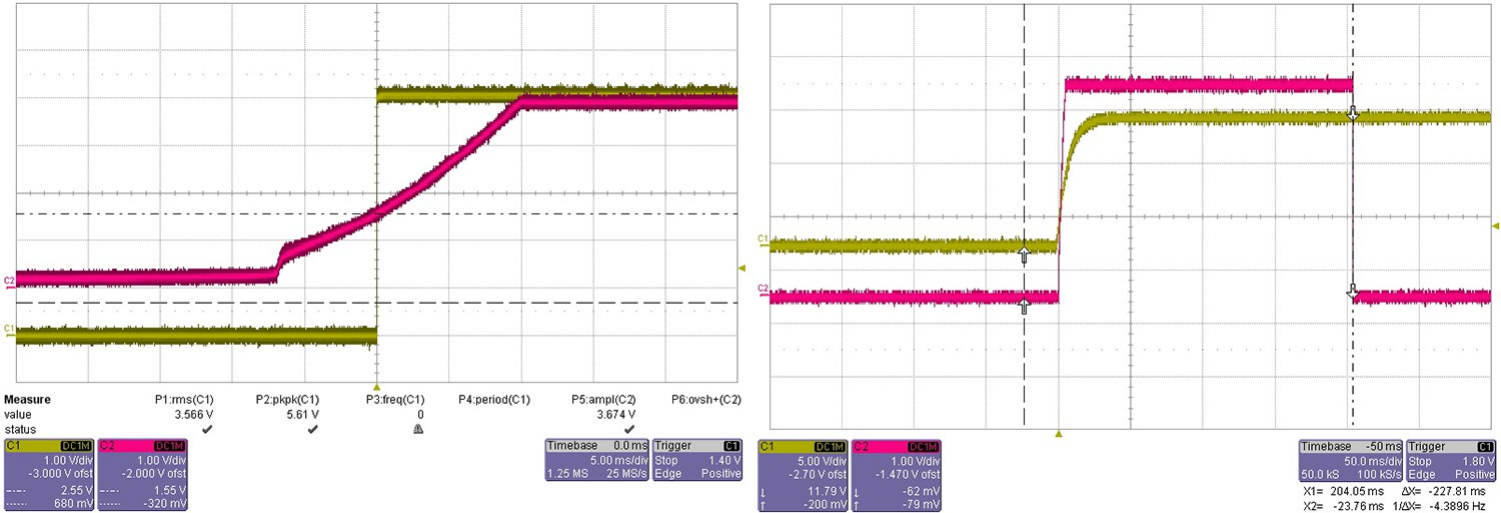
(a) Waveform of liquid level detection signal (the liquid to be tested is filled with reagent bottle). (b) Waveform of liquid level detection signal (the volume of liquid to be tested is 200uL).

The test results are shown in Fig 24(A) and 24(B).

In Fig 24(A) and 24(B), the yellow waveform is the liquid level detection signal output by the liquid level detection circuit board, and the red waveform is the analog liquid level signal. When the probe does not contact the liquid level, the liquid level detection circuit board outputs low level, and when the probe contacts the liquid level, it outputs high level. After repeated tests, it works well and the test is passed.

### 4.7 Test of anti-collision detection function

The test requirements are as follows: before the accidental collision of the probe during the movement, the detection circuit outputs high level, and after the collision, the detection circuit outputs low level. The output level meets the TTL logic voltage level requirements.

**Fig 25:**
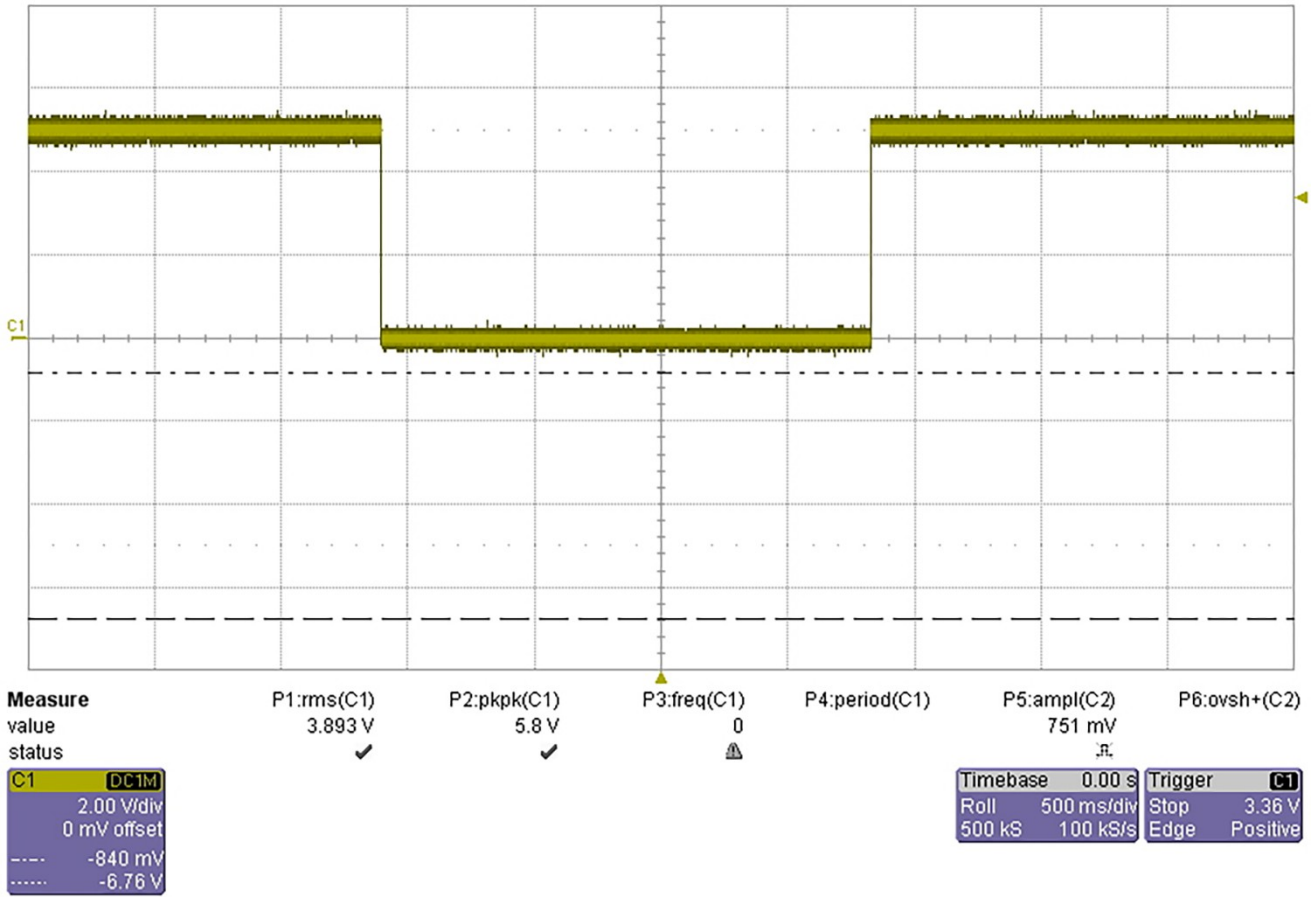
Waveform of anti-collision detection test.

The test results are shown in Fig 25.

Block the transmitting hole of the anti-collision sensor with a baffle, and the detection circuit outputs a high level; The baffle leaves the sensor transmitting hole and the detection circuit outputs low level. The output level meets the TTL logic voltage level requirements, and the test is passed.

## 5 Conclusions

This paper presents a design method of accurate liquid level detection circuit based on variable capacitance principle. In this circuit, the double-layer sampling probe is connected to the circuit as a capacitance sensor. With the PLL chip HEF4046BT as the core, the capacitance change before and after the probe contacts the liquid level is transformed into the analog voltage output of the PLL. After filtering, amplification and A/D conversion, the combination of slope detection and amplitude detection is used to analyze, judge and output the liquid level detection signal.

The experimental test shows that the system can quickly and accurately detect the liquid level in the case of large and small liquid volume, and the operation is stable and reliable. The circuit can be used in the automatic sampling module of medical testing instruments such as automatic biochemical analyzers. It is of great significance to improve the accuracy and stability of measurement results.

In order to further improve the system performance and improve the sensitivity of liquid level detection, the following measures can be taken:①Improving the processing speed of microcontroller;②Further increasing the capacitance change of the probe;③Optimizing the equilibrium recovery time of analog circuit of liquid level detection circuit board. In the future research, it is necessary to further improve the function of the system, optimize the structure of the system, and realize a more accurate, reliable and fast response liquid level detection system.
